# Author Correction: Genome-wide association study of self-reported walking pace suggests beneficial effects of brisk walking on health and survival

**DOI:** 10.1038/s42003-020-01447-6

**Published:** 2020-11-10

**Authors:** Iain R. Timmins, Francesco Zaccardi, Christopher P. Nelson, Paul W. Franks, Thomas Yates, Frank Dudbridge

**Affiliations:** 1 Department of Health Sciences, University of Leicester, Leicester, UK; 2 Diabetes Research Centre, University of Leicester, Leicester, UK; 3 Department of Cardiovascular Sciences, University of Leicester, Leicester, UK; 4 NIHR Leicester Biomedical Research Centre, University Hospitals of Leicester NHS Trust & University of Leicester, Leicester, UK; 5 Department of Clinical Sciences, Lund University, Lund, Sweden

**Keywords:** Risk factors, Genome-wide association studies, Metabolic diseases

Correction to: *Communications Biology* 10.1038/s42003-020-01357-7, published online 30 October 2020.

In the original published version of the article, the middle initial for author Paul W. Franks was incorrectly omitted. The error has been corrected in the PDF and HTML versions of the article.

