# Supplementary material for: Genome-wide association study of self-reported walking pace suggests beneficial effects of brisk walking on health and survival
Source: Commun Biol. 2020 Oct 30;3:634. doi: 10.1038/s42003-020-01357-7 (PMC7599247; doi:10.1038/s42003-020-01357-7)
Supplement: Supplementary file 1 — Supplementary Information [file 42003_2020_1357_MOESM1_ESM.pdf]

Supplementary Information

Contents

Supplementary Note 1 2

Supplementary Figure 1 5

Supplementary Figure 2 5

References 6

Supplementary Note 1

Observed and latent variable scales for ordinal traits

For convenience we present here a derivation of the heritability on the liability scale, originally shown by Gianola (1979).

We have observed self-reported walking pace as an ordered categorical variable with responses "slow", "average" and "brisk" pace which are be coded as 0, 1 and 2.

More generally we can consider a trait observed with ordered categories of response $y = 0, 1, \ldots, N$. Suppose an underlying latent variable z captures the true trait on a continuous scale as shown:

$$y = \begin{cases} 0 & \text{if } z \leq \mu_1 \\ 1 & \text{if } \mu_1 < z \leq \mu_2 \\ 2 & \text{if } \mu_2 < z \leq \mu_3 \\ \vdots & \vdots \\ N & \text{if } \mu_N < z \end{cases}$$

We make the further assumption that the true trait has a standard normal distribution $z \sim N(0,1)$. Let y take on the values 0, 1 …, N with frequencies π_i.

On the observed scale, the trait is assumed to have independent genetic u_i and environmental effects e_i. For individual i we have $y_i = c + u_i + e_i$, where c denotes an intercept, and where $u_i \sim N(0, \sigma_u^2)$ and $e_i \sim N(0, \sigma_e^2)$. The heritability h_{obs}^2 on the observed scale is the proportion of phenotypic variance explained by genetic factors $h_{obs}^2 = \sigma_u^2/\text{Var}[Y]$, where $\text{Var}[Y] = \sum_{k=1}^{N} k^2 \pi_k - (\sum_{k=1}^{N} k\pi_k)^2$.

On the latent variable scale we similarly have independent genetic g_i and environmental ε_i effects where $z_i = g_i + \varepsilon_i$ (no intercept is needed here since z has a standard normal distribution). The genetic variance in this case can be described by $g_i \sim N\left(0, \sigma_g^2\right)$. The heritability on the latent variable scale is simply $h_l^2 = \sigma_g^2$.

It has been shown by Dempster and Lerner (1950) that the genetic values on the two scales are related by a linear scale $u = \alpha + \beta g$. Moreover $\beta = \text{Cov}[y, g]/\text{Var}[g]$. To evaluate β we derive a few results.

Firstly, since $g \sim N(0, \sigma_g^2)$, $z \sim N(0,1)$ and $z = g + \varepsilon$, it is a standard conditional probability result for normal distributions that for an arbitrary t, $\mathbb{E}[g|z = t] = t\sigma_g^2$. Let φ, Φ denote the standard normal density and cumulative distribution functions, respectively. A standard normal distribution truncated to between μ_i and μ_{i+1} has density function $\varphi(t)/(\Phi(\mu_{i+1})\text{-}\Phi(\mu_i))$ and mean $(\varphi(\mu_i)\text{-}\varphi(\mu_{i+1}))/(\Phi(\mu_{i+1})\text{-}\Phi(\mu_i))$. To determine β, first we have:

$$\begin{aligned} \mathbb{E}[g|y = i] &= \mathbb{E}[g|\ \mu_i < z \leq \mu_{i+1}] \\ &= \int_{\mu_i}^{\mu_{i+1}} \mathbb{E}[g|\ z = t] \frac{\varphi(t)}{\Phi(\mu_{i+1})\text{-}\Phi(\mu_i)} dt \\ &= \int_{\mu_i}^{\mu_{i+1}} t\sigma_g^2 \frac{\varphi(t)}{\Phi(\mu_{i+1})\text{-}\Phi(\mu_i)} dt \\ &= \sigma_g^2 \int_{\mu_i}^{\mu_{i+1}} t \frac{\varphi(t)}{\Phi(\mu_{i+1})\text{-}\Phi(\mu_i)} dt \\ &= \sigma_g^2 \mathbb{E}[z|\ \mu_i < z \leq \mu_{i+1}] \end{aligned}$$

$$= \sigma_g^2 \frac{\varphi(\mu_i)\text{-}\varphi(\mu_{i+1})}{\Phi(\mu_{i+1})\text{-}\Phi(\mu_i)}$$

Conditioning on y (where we define $\mu_0 = -\infty$, $\mu_{N+1} = \infty$ and $\varphi(\mu_0) = \varphi(\mu_{N+1}) = 0$):

$$\begin{aligned}
\mathbb{E}[yg] &= \sum_{i=0}^{N} \mathbb{E}[yg|y = i]\mathbb{P}(y = i) \\
&= \sum_{i=0}^{N} i\mathbb{E}[g|y = i]\mathbb{P}(y = i) \\
&= \sum_{i=0}^{N} i\sigma_g^2 \frac{\varphi(\mu_i)\text{-}\varphi(\mu_{i+1})}{\Phi(\mu_{i+1})\text{-}\Phi(\mu_i)} \mathbb{P}(\, \mu_i < z \leq \mu_{i+1}) \\
&= \sum_{i=0}^{N} i\sigma_g^2 (\varphi(\mu_i)\text{-}\varphi(\mu_{i+1})) \\
&= \sum_{i=1}^{N} \sigma_g^2 \varphi(\mu_i)
\end{aligned}$$

Next,

$$\begin{aligned}
\beta &= \frac{\mathrm{Cov}[y, g]}{\mathrm{Var}[g]} \\
&= \frac{\mathbb{E}[yg] - \mathbb{E}[y]\mathbb{E}[g]}{\sigma_g^2} \\
&= \frac{\mathbb{E}[yg]}{\sigma_g^2} \\
&= \sum_{i=1}^{N} \varphi(\mu_i)
\end{aligned}$$

We also have:

$$\begin{aligned}
\sigma_u^2 &= \mathrm{Var}[u] \\
&= \mathrm{Var}[\alpha + \beta g] \\
&= \beta^2 \mathrm{Var}[g] \\
&= \beta^2 \sigma_g^2
\end{aligned}$$

Finally, linking these steps together:

$$\begin{aligned}
h_o^2 &= \frac{\sigma_u^2}{\mathrm{Var}[Y]} \\
&= \frac{\beta^2 \sigma_g^2}{\mathrm{Var}[Y]} \\
&= \frac{\beta^2 h_l^2}{\mathrm{Var}[Y]} \\
&= h_l^2 \frac{\left(\sum_{i=1}^{N} \varphi(\mu_i)\right)^2}{\mathrm{Var}[Y]}
\end{aligned}$$

This demonstrates how to convert between the observed and latent variable scales for an ordinal categorical variable.

In this study, we have 3 categories for self-reported walking pace, so $N = 2$. They are coded as $y = 0, 1$ and 2 for slow, average and brisk walkers. There are 34,567 slow and 236,026 average paced walkers, so $\varphi(\mu_1) = \varphi\left(\Phi^{-1}\left(\frac{34{,}567}{450{,}967}\right)\right) = 0.144$ and $\varphi(\mu_2) = \varphi\left(\Phi^{-1}\left(\frac{34{,}567+236{,}026}{450{,}967}\right)\right) = 0.386$. Also, $\text{Var}[Y] = 0.372$, which results in $h_o^2 = 0.756\ h_l^2$.

Supplementary Figure 1

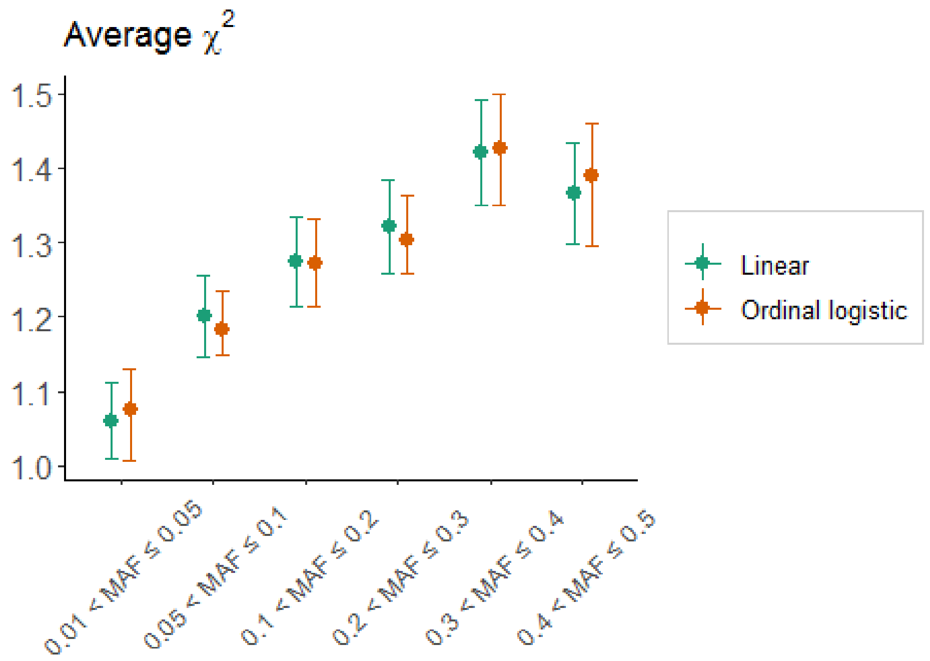

Supplementary Figure 1: Distribution of average χ^2 values for a random sample of 1,000 SNPs in 6 minor allele frequency bins. The points and error bars represent the mean and ±1 s.e.

Supplementary Figure 2

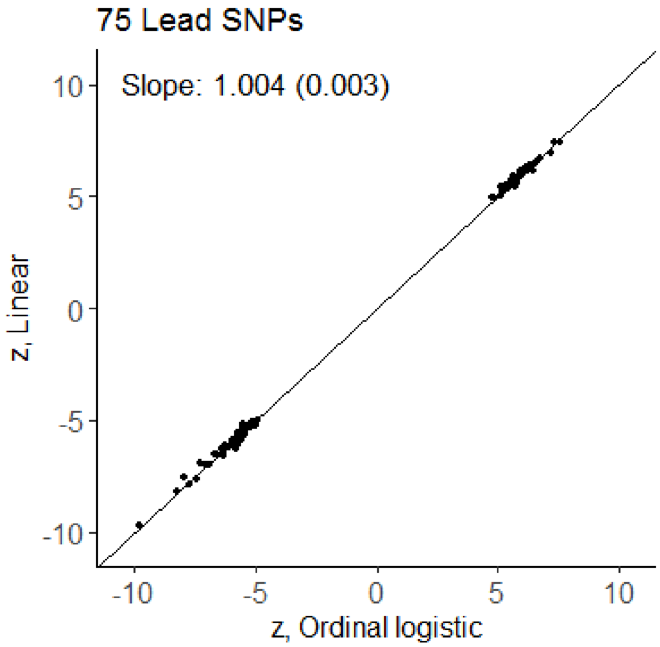

Supplementary Figure 2: We plotted standardized effect sizes (z-scores) for the 75 genome-wide significant lead SNPs under a linear model (phenotype coded 0,1,2) versus ordinal logistic regression.

References

1. Gianola D. Heritability of polychotomous characters. Genetics. 1979;93(4):1051.
2. Dempster E.R., Lerner I.M. Heritability of threshold characters. Genetics. 1950;35:212–236.
