# Supplementary material for: Genome-wide association study of self-reported walking pace suggests beneficial effects of brisk walking on health and survival
Source: Commun Biol. 2020 Oct 30;3:634. doi: 10.1038/s42003-020-01357-7 (PMC7599247; doi:10.1038/s42003-020-01357-7)
Supplement: Supplementary file 3 — Description of Additional Supplementary Files [file 42003_2020_1357_MOESM3_ESM.pdf]

Description of Additional Supplementary Files

File Name: Supplementary Data 1-11

Description:

Supplementary Data 1: Cohort characteristics, by self-reported walking pace.

Supplementary Data 2: 75 Independent lead SNPs for self-reported walking pace

Supplementary Data 3: Functional annotation of SNPs in the 70 risk loci in LD (r2>0.6) with the independent significant SNPs.

Supplementary Data 4: Catalogue of previously reported GWAS associations from the NCBI database for self-reported walking pace genomic loci

Supplementary Data 5. Self-reported walking pace genes implicated by positional mapping and/or eQTL mapping.

Supplementary Data 6: Genes associated with self-reported walking pace using genome- wide gene -based analysis in MAGMA.

Supplementary Data 7: MAGMA gene-property analysis for self-reported walking pace.

Supplementary Data 8: Genetic correlations

Supplementary Data 9: Mendelian randomisation

Supplementary Data 10: Mendelian randomisation excluding adiposity related SNPs

Supplementary Data 11: Multivariable Mendelian Randomisation, adjusting for BMI
