# Supplementary material for: Genome-wide association study of self-reported walking pace suggests beneficial effects of brisk walking on health and survival
Source: Commun Biol. 2020 Oct 30;3:634. doi: 10.1038/s42003-020-01357-7 (PMC7599247; doi:10.1038/s42003-020-01357-7)
Supplement: Supplementary file 5 — Reporting Summary [file 42003_2020_1357_MOESM5_ESM.pdf]

nature research

Corresponding author(s): Frank Dudbridge

Last updated by author(s): Jun 4, 2020

Reporting Summary

Nature Research wishes to improve the reproducibility of the work that we publish. This form provides structure for consistency and transparency in reporting. For further information on Nature Research policies, see our Editorial Policies and the Editorial Policy Checklist.

Statistics

For all statistical analyses, confirm that the following items are present in the figure legend, table legend, main text, or Methods section.

n/a	Confirmed	
☐	☒	The exact sample size (*n*) for each experimental group/condition, given as a discrete number and unit of measurement
☐	☒	A statement on whether measurements were taken from distinct samples or whether the same sample was measured repeatedly
☐	☒	The statistical test(s) used AND whether they are one- or two-sided<br>*Only common tests should be described solely by name; describe more complex techniques in the Methods section.*
☐	☒	A description of all covariates tested
☐	☒	A description of any assumptions or corrections, such as tests of normality and adjustment for multiple comparisons
☐	☒	A full description of the statistical parameters including central tendency (e.g. means) or other basic estimates (e.g. regression coefficient) AND variation (e.g. standard deviation) or associated estimates of uncertainty (e.g. confidence intervals)
☐	☒	For null hypothesis testing, the test statistic (e.g. *F*, *t*, *r*) with confidence intervals, effect sizes, degrees of freedom and *P* value noted<br>*Give P values as exact values whenever suitable.*
☒	☐	For Bayesian analysis, information on the choice of priors and Markov chain Monte Carlo settings
☒	☐	For hierarchical and complex designs, identification of the appropriate level for tests and full reporting of outcomes
☐	☒	Estimates of effect sizes (e.g. Cohen's *d*, Pearson's *r*), indicating how they were calculated

Our web collection on statistics for biologists contains articles on many of the points above.

Software and code

Policy information about availability of computer code

Data collection	Data were downloaded from UK Biobank using their provided software tool ukbgene
Data analysis	Data were analysed using Rv3.3, STATAv16.0 and custom software BOLT-LMMv2.3.2, PLINKv1.9, FUMA, KING, LDSCv1.01, ORDINALGWAS.JL, PRSICEv2.2.3

For manuscripts utilizing custom algorithms or software that are central to the research but not yet described in published literature, software must be made available to editors and reviewers. We strongly encourage code deposition in a community repository (e.g. GitHub). See the Nature Research guidelines for submitting code & software for further information.

Data

Policy information about availability of data

All manuscripts must include a data availability statement. This statement should provide the following information, where applicable:

- Accession codes, unique identifiers, or web links for publicly available datasets
- A list of figures that have associated raw data
- A description of any restrictions on data availability

UK Biobank data are available through a procedure described at http://www.ukbiobank.ac.uk/using-the-resource/. Genetic associations with BMI are available from the GIANT consortium at https://portals.broadinstitute.org/collaboration/giant/images/1/15/SNP_gwas_mc_merge_nogc.tbl.uniq.gz

Field-specific reporting

Please select the one below that is the best fit for your research. If you are not sure, read the appropriate sections before making your selection.

☒ Life sciences ☐ Behavioural & social sciences ☐ Ecological, evolutionary & environmental sciences

For a reference copy of the document with all sections, see nature.com/documents/nr-reporting-summary-flat.pdf

Life sciences study design

All studies must disclose on these points even when the disclosure is negative.

Sample size	The study used the UK Biobank resource which is larger than any genetic study of walking pace previously performed, and has yielded large numbers of genetic associations with other complex traits.
Data exclusions	Analysis was restricted to subjects of European ancestry as defined by K-means clustering of genetic principal components.
Replication	We approached two independent cohorts, Whitehall II and the Nurses Health Study, with a view to replicating our results. However neither cohort was responsive to repeated requests for co-operation. We did not identify any other cohort with a similar phenotype recorded in a comparable population.
Randomization	We controlled for age, sex, genotyping array and 20 principal components of genetic ancestry.
Blinding	This was an observational study of previously collected data and blinding was not required.

Reporting for specific materials, systems and methods

We require information from authors about some types of materials, experimental systems and methods used in many studies. Here, indicate whether each material, system or method listed is relevant to your study. If you are not sure if a list item applies to your research, read the appropriate section before selecting a response.

Materials & experimental systems

n/a	Involved in the study	
☒	☐	Antibodies
☒	☐	Eukaryotic cell lines
☒	☐	Palaeontology and archaeology
☒	☐	Animals and other organisms
☐	☒	Human research participants
☒	☐	Clinical data
☒	☐	Dual use research of concern

Methods

n/a	Involved in the study	
☒	☐	ChIP-seq
☒	☐	Flow cytometry
☒	☐	MRI-based neuroimaging

Human research participants

Policy information about studies involving human research participants

Population characteristics	The UK Biobank consists of UK residents aged between 40 and 69 at recruitment from the year 2010, of predominantly white European ancestry. In the study sample the mean age was 56.8 and proportion of males was 45.7%.
Recruitment	Participants were invited by letter and self-selected into the study. Research has demonstrated lower rates of some diseases, such as type 2 diabetes, in the UK Biobank compared to the general population, and higher socio-economic category on average. This may create selection bias in this study if direct genetic effects of walking pace are confounded by common causes of study participation and walking pace. We conditioned analyses on BMI to allow for this possibility.
Ethics oversight	The UK Biobank study has ethics approval from the North West National Research Ethics Committee (REC reference 11/NW/0274).

Note that full information on the approval of the study protocol must also be provided in the manuscript.
